# Changing Reimbursement Criteria on Anti-VEGF Treatment Patterns Among Wet Age-Related Macular Degeneration and Diabetic Macular Edema Patients: An Interrupted Time Series Analysis

**DOI:** 10.34172/ijhpm.8210

**Published:** 2024-07-07

**Authors:** Yu-Wen Huang, Lin-Chieh Meng, Li-Jiuan Shen, Chih-Fen Huang, Liang-Kung Chen, Fei-Yuan Hsiao

**Affiliations:** ^1^School of Pharmacy, National Taiwan University, Taipei, Taiwan.; ^2^Graduate Institute of Clinical Pharmacy, College of Medicine, National Taiwan University, Taipei, Taiwan.; ^3^Department of Pharmacy, National Taiwan University Hospital, Taipei, Taiwan.; ^4^Center for Geriatrics and Gerontology, Taipei Veterans General Hospital, Taipei, Taiwan.; ^5^Center for Healthy Longevity and Aging Sciences, National Yang Ming Chiao Tung University, Hsinchu, Taiwan; ^6^Taipei Municipal Gan-Dau Hospital (Managed by Taipei Veterans General Hospital), Taipei, Taiwan.

**Keywords:** Anti-Vascular Endothelial Growth Factor, Wet Age-Related Macular Degeneration, Diabetic Macular Edema, Reimbursement Policies, Interrupted Time Series Analysis

## Abstract

**Background:**

To evaluate the impact of reimbursement criteria change on the utilization pattern of anti-vascular endothelial growth factor (anti-VEGF) among patients with wet age-related macular degeneration (wAMD) and diabetic macular edema (DME) separately in Taiwan.

**Methods:**

An interrupted time series analysis (ITSA) was performed using Taiwan’s National Health Insurance (NHI) database, and patients with wAMD or DME diagnosis at the first injection of anti-VEGF agents was identified from 2011 to 2019. The outcome of interest was treatment gaps between injections of anti-VEGF. This outcome was retrieved quarterly, and the study period was divided into three phases in wAMD (two criteria changed in August 2014 [intervention] and December 2016 [intervention]) and two phases in DME (three consecutive criteria changed in 2016 [intervention]). Segmented regression models adjusted for autocorrelation were used to estimate the change in level and the change in slope of the treatment gaps between each anti-VEGF injection.

**Results:**

The treatment gaps between each anti-VEGF injection decreased from 2011 to 2019. The cancellation of the annual three needles limitation was associated with significantly shortened treatment gaps between the third and fourth needles (wAMD change in level: -228 days [95% CI -282, -173], DME change in level: -110 days [95% CI -141, -79]). The treatment gap between the fifth and sixth needles revealed a similar pattern but without significant change in DME patients. Other treatment gaps revealed considerable change in slopes in accordance with criteria changes.

**Conclusion:**

This is the first nationwide study using ITSA to demonstrate the impact of reimbursement policy on treatment gaps between each anti-VEGF injection. After canceling the annual limitation, we found that the treatment gaps significantly decreased among wAMD and DME patients. The shortened treatment gaps might further link to better visual outcomes according to previous studies. The different impacts from criteria changes can assist future policy shaping. Future studies were warranted to explore whether such changes are associated with the benefits of visual effects.

## Background

**Key Messages**

**Implications for policy makers**

- There was a decreasing trend in the treatment gap between each anti-vascular endothelial growth factor (anti-VEGF) injection for both wet age-related macular degeneration (wAMD) and diabetic macular edema (DME) patients from 2011 to 2019.
- The removal of the annual needles limitation resulted in a significant change in level in the treatment gap between the third and fourth needles in both indications.
- A future focus should be on exploring whether such changes are associated with the benefits of visual effects.

**Implications for the public**

Anti-VEGF therapies have been reimbursed by the National Health Insurance (NHI) in Taiwan for over a decade, ensuring the growing population has access to effective treatments. The reimbursement criteria have been revised several times, in various ways, impacting clinical practice. This study aimed to assess how these changes influenced the prescription patterns of anti-vascular endothelial growth factor (anti-VEGF) agents in both wet age-related macular degeneration (wAMD) and diabetic macular edema (DME) patients. Our study results showed a decreasing trend in the treatment gap between each needle from 2011 to 2019, regardless of the indications. When the limitation on the number of injections was loosened from an annual limit to application limit, there was a significant change in level in the treatment gap between the third and fourth needles for both wAMD and DME. The findings in the present study could help both clinical practitioners and policy-makers understand the real-world impact on treatment patterns resulting from each criteria change.

With the worldwide growth of the older adult population, age-related ophthalmology diseases have become a significant concern. Among these diseases, wet age-related macular degeneration (wAMD) is one of the most common and has exhibited a global prevalence of 8.7%, leading to an increased disease burden.^1^ In Taiwan, a study has shown that individuals over 65 have a prevalence of 7.3% for late age-related macular degeneration.^2^ Additionally, diabetic macular edema (DME), another frequent ophthalmology disease, has reached a global prevalence of 7.5% among the diabetes population.^3^ Both wAMD and DME can result in a decline in patients’ visual acuity and even blindness, raising social costs.^4-6^

 In recent years, treatments for ophthalmic diseases have significantly advanced, and intravitreal anti-vascular endothelial growth factor (anti-VEGF) treatments have been proven to be an effective first-line therapy for wAMD and DME.^7,8^ According to previous trials, three initial monthly doses of anti-VEGF are crucial in reaching sufficient efficacy.^9,10^ In local studies, the effectiveness of three consecutive injections was also observed.^11,12^ Nevertheless, uncertainty persists regarding the optimal frequency and number of injections of anti-VEGF for treating wAMD or DME. A previous study in Taiwan has demonstrated a positive correlation between the number of injections and visual outcomes for DME patients.^13^ Another study recommended administering eight to nine doses of anti-VEGF in the first treatment year for Asian DME patients.^14^

 As the number of anti-VEGF injections increases, so do the tremendous drug costs associated with them. This further complicates the question of the optimal frequency and number of injections, particularly within single-payer healthcare systems such as Taiwan’s.^15^ Taiwan’s National Health Insurance (NHI) is a mandatory, government-run health insurance program that covers more than 99% of the entire population (approximately 24 million) in Taiwan. As an insurer with limited resources, NHI must evaluate each new health technology (including drugs) and decide whether they should be included in their reimbursement scheme. In some instances, reimbursement for certain drugs may be based on specific criteria, and anti-VEGF agents in Taiwan fall into this category.

 For example, ranibizumab was initially authorized for reimbursement as the first anti-VEGF for wAMD in Taiwan in 2011. Subsequently, in 2013, it was further approved for DME. Over the past decade, the reimbursement criteria for ranibizumab have undergone multiple revisions, particularly concerning the number and frequency of injections, driven by diverse considerations. Notably, anti-VEGF therapy for wAMD or DME can be reimbursed via pre-authorization, whereby only limited injections (wAMD: three injections; DME: five injections) of anti-VEGF are reimbursed for the first year. Additional injections (wAMD: four injections; DME: three injections) may be approved, provided there is supporting evidence of patient improvement with the previous anti-VEGF therapy. The annual limitations were canceled in 2014 and 2016 for wAMD and DME, respectively. Prior to 2016, these injections were required to be administered within two years, whereas afterward, they could be administered within a five-year timeframe. Such changes in reimbursement criteria have the potential to impact the prescribing patterns of anti-VEGF agents and the clinical outcomes of the affected patients.

 Hence, this study aims to employ interrupted time series analysis (ITSA)^16^ to assess the effects of changes in reimbursement criteria on the prescription patterns of anti-VEGF agents.

## Methods

###  Data Source 

 This is a retrospective, nationwide study using the NHI data provided by the Health and Welfare Data Science Center (HWDC) of the Ministry of Health and Welfare, Taiwan.

 The NHI is a government-run, single-payer, compulsory health insurance system launched in 1995. The NHI system provides comprehensive coverage for outpatient and inpatient services, medications, diagnostic tests, procedures, and surgeries for its beneficiaries. Under this system, detailed healthcare information of each beneficiary, including demographics, healthcare utilizations, diagnoses, procedures, and drug prescriptions, is well documented.^17^ Due to the longitudinal data spanning more than 15 years and the 99% coverage of Taiwan’s population of more than 24 million, the NHI data has been used extensively to generate real-world evidence regarding nationwide utilization patterns of drugs.^18-21^

###  Study Subjects

 The study identified patients who were prescribed anti-VEGF agents and categorized them into two distinct cohorts: wAMD, which was defined as a medical diagnosis of International Classification of Diseases, 9th Revision, Clinical Modification (ICD-9-CM) codes 362.50, 362.52, 362.57 or International Classification of Diseases, 10th Revision, Clinical Modification (ICD-10-CM) codes H35.30, H35.32, H35.361, H35.362, H35.363, H35.369) and DME, which was defined as a diagnosis of ICD-9-CM codes 362.07, 250.5, 362.01, 362.02, 362.03, 362.04, 362.05, 362.06, 250.0x +362.53, 250.0x +362.83 or ICD-10-CM codes E10.31x, E10.321x, E10.331x, E10.341x, E10.351x, E11.31x, E11.321x, E11.331x, E11.341x, E11.35x, E13.31x, E13.321x, E13.331x, E13.341x, E13.351x. The stratification into the respective subgroups was based on the diagnosis rendered on the same date of anti-VEGF treatment initiation. The two indications would be analyzed separately based on their indication-specific reimbursement criteria and the results would be demonstrated by indications.

###  Use of Anti-VEGF and Other Ophthalmological Agents

 During the study period, two anti-VEGF agents were reimbursed by the NHI, ranibizumab (Anatomical Therapeutic Chemical [ATC code S01LA04] and aflibercept [ATC code S01LA05]). Bevacizumab was not included in this study due to its off-label use. Other ophthalmological agents, including antibiotics and steroids, were also captured in this study.

**Figure 1:**
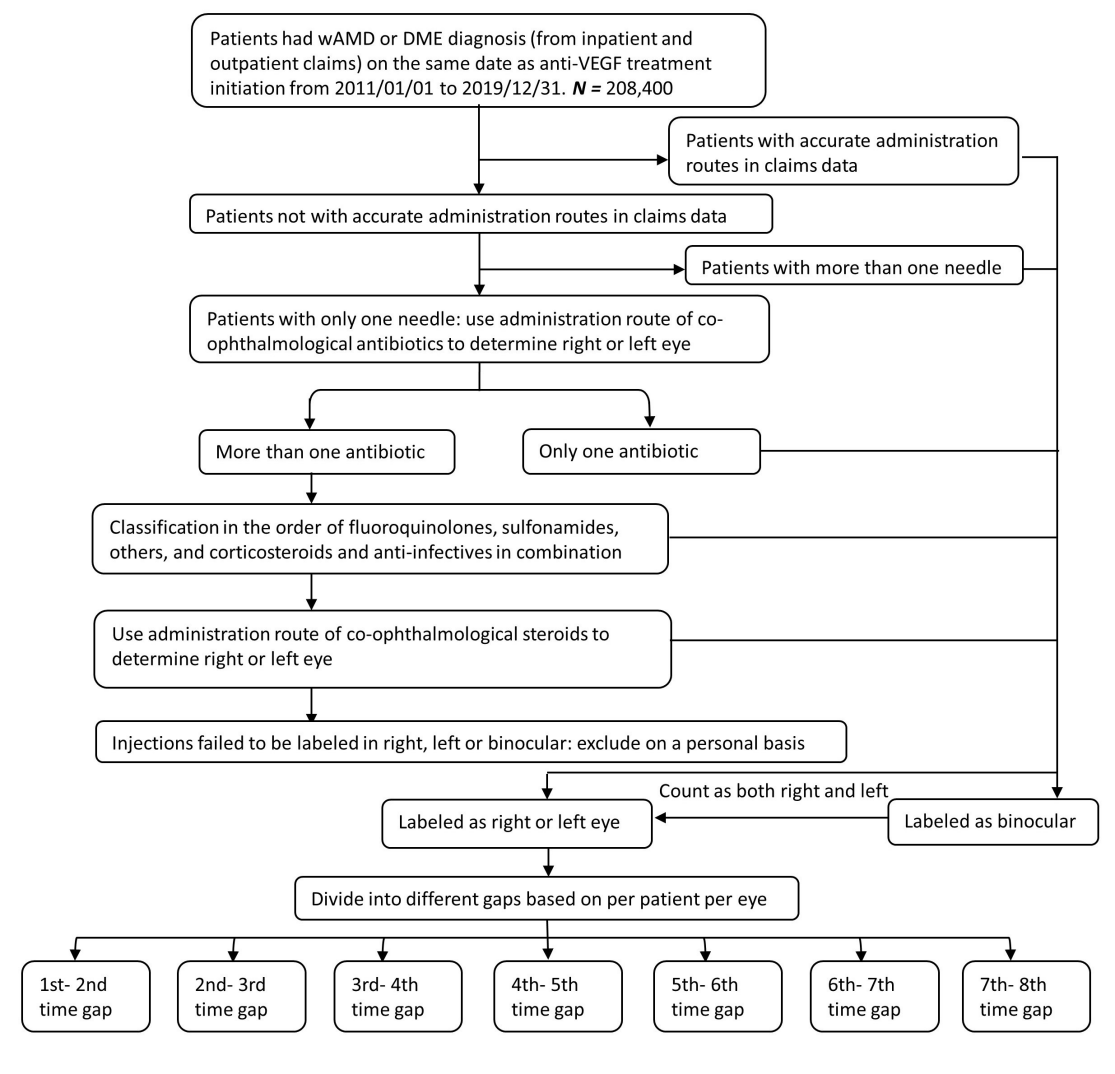
Algorithm for Classification of the Laterality of the Eye. Abbreviations: wAMD, wet age-related macular degeneration; DME, diabetic macular edema; anti-VEGF, anti-vascular endothelial growth factor.

 To capture the treatment gaps between anti-VEGF injections in claims data, we implemented an algorithm (Figure 1) that considers the injection site and other pertinent ophthalmological agents. Initially, we identified patients whose administration routes were recorded in the claims data, designated as “OD” for the right eye, “OS” for the left eye, or “OU” for both eyes and assigned injection sites accordingly. Patients receiving more than one needle were classified as binocular users based on the total number of injections documented in the claims data. In instances where the administration site for anti-VEGF therapy was not specified, we utilized the administration routes of other ophthalmological medications, including antibiotics and steroids, frequently prescribed to prevent injection-related infections, to label the injection site. Hence, individual patient data can be tagged with the laterality and sequential order from the first to the last injection time. Patients whose anti-VEGF administration site could not be determined were excluded from the analysis. Lastly, the treatment gaps (ie, time to next injection) between anti-VEGF injections were computed according to the tags (Supplementary file 1, Figure S1). For instance, a wAMD patient received first anti-VEGF in January 2015 at right eye and second anti-VEGF in February 2015 at the same eye would be considered as one data point for first to second treatment gap in 2015Q1 wAMD population.

###  Demographic and Clinical Characteristics

 Demographic variables, such as age (categorized as ≤50 years, 51-64 years, 65-74 years, 75-84 years, or ≥85 years), sex, and the calendar year of diagnosis, were recorded at the time of the first anti-VEGF injection. To evaluate individual patients’ baseline health status, the Charlson Comorbidity Index,^22^ including myocardial infarction, congestive heart failure, peripheral vascular disease, cerebrovascular disease, dementia, chronic pulmonary disease, rheumatologic disease, peptic ulcer disease, mild liver disease, diabetes, diabetes with chronic complications, hemiplegia or paraplegia, renal disease, malignancy (including leukemia and lymphoma), moderate or severe liver disease, metastatic solid tumor, and acquired immunodeficiency syndrome, was calculated within one year before the date of injection using NHI claim data. In addition, laterality (right, left, or binocular) ophthalmic comorbidities, including retinal neovascularization, central retinal vein occlusion, branch retinal vein occlusion, and the use of other ophthalmological medications, were also evaluated during the baseline period.

###  Statistical Analyses

 An interrupted time-series analysis (ITSA)^16^ was applied to evaluate the impact of changes in reimbursement criteria on quarterly prescription patterns of anti-VEGF among wAMD and DME patients separately. ITSA is a quasi-experimental method that can provide the pre-existing trends of treatment gaps and estimate changes resulting from the revisions in reimbursement criteria. Specifically, we used segmented regression models to assess both the level and change in slopes in the treatment gaps (time to next injection) of anti-VEGF on a quarterly base.

 In this study, we specifically focus on revisions in reimbursement criteria of anti-VEGFs for wAMD and DME between 2011 and 2020 (Supplementary file 1, Table S1). To be brief, anti-VEGF therapy for wAMD or DME can be reimbursed via pre-authorization, whereby only limited injections (wAMD: three injections; DME: five injections) of anti-VEGF are reimbursed for the first year. Additional injections (wAMD: four injections; DME: three injections) may be approved, provided there is supporting evidence of patient improvement with the previous anti-VEGF therapy. The annual limitations were canceled in 2014 and 2016 for wAMD and DME, respectively. Prior to 2016, these injections were required to be administered within two years, whereas afterward, they could be administered within a five-year timeframe.

 The segmented regression models for wAMD included two interventions, with a total of 14 data points before the criteria change (2011 Q1–2014 Q2), 7 data points after the first criteria change (2014 Q4–2016 Q3), and 12 data points following the second criteria change (2017 Q1–2019 Q4). Notably, the two data points in the transition period (2014 Q3 and 2016 Q4) were excluded from the analysis. Similarly, the segmented regression models for DME featured one intervention, with 12 data points before the criteria change (2013 Q1–2015 Q4) and 12 data points after the criteria change (2017 Q1–2019 Q4). The data points within the transition period (2016 Q1–Q4) were also excluded.

 The segmented regression model employed in this study comprised several terms^23^ to estimate the (*a*) intercept, representing the level at the start of the preregulation period; (*b*) slope, representing the trend in the preregulation period; (*c*) change in level, representing the absolute effects of immediate change at the beginning of the post-regulation period; and (*d*) change in slope, representing the absolute effects of difference between the pre-and post-regulation slopes. To account for autocorrelation, the Durbin-Watson test was utilized and, if significant, adjusted in the models.^24^ To reveal the short-term impact of the reimbursement criteria change, we compared the estimated post-regulation treatment gaps with the predicted treatment gaps in 2014 Q4 and 2017 Q1 for wAMD and in 2017 Q1 for DME. The predicted treatment gaps are computed utilizing the baseline level and baseline trend, assuming no changes in reimbursement criteria. In contrast, the estimated treatment gaps comprise the actual data points collected after the policy change. The disparities between estimated and predicted treatment gaps can thus signify immediate policy impacts, reflecting short-term effects. To evaluate the long-term effect of the regulations, the changes in slopes were reported in 2016 Q3 and 2019 Q4 for wAMD and in 2019 Q4 for DME.

 All the analyses were employed using SAS, version 9.4 (SAS Institute Inc., Cary, NC, USA). A two-sided *P *value was used, with *P*< .05 considered statistically significant.

## Results

###  Demographic and Clinical Characteristics

Table presents an overview of the demographic characteristics of the study participants, classified according to the indication for treatment (wAMD or DME) and type of anti-VEGF received (ranibizumab or aflibercept). In total, 22 792 patients were included in this study, with 15 722 (69.0%) being treated for wAMD and 7070 (31.0%) for DME. The mean age of patients with wAMD was approximately 70 years, irrespective of the anti-VEGF used, while those with DME were younger, with an average age of 62 years. A higher proportion of males were observed in all groups. We also noted a gradual decline in ranibizumab usage over the 9-year period, with a concomitant increase in the number of aflibercept users, regardless of indication.

**Table:** Baseline Characteristics of wAMD and DME Patients Using Anti-vascular Endothelial Growth Factor Agents

	**wAMD**			**DME**		
	**Ranibizumab** ** (n = 6825)**	**Aflibercept** **(n = 8897)**	***P*** ** Value**	**Ranibizumab** ** (n = 4768)**	**Aflibercept** **(n = 2302)**	***P*** ** Value**
Age (y), mean (SD)	70.61(11.99)	70.46(11.36)	.42	62.48 (10.46)	62.87 (11.11)	.16
Age group (y), No. (%)						
≤50	344 (5.04)	355 (3.99)	<.01	565 (11.85)	286 (12.42)	<.01
51-64	1709 (25.04)	2205 (24.78)		2172 (45.55)	954 (41.44)	
65-74	1998 (29.27)	2916 (32.78)		1461 (30.64)	750 (32.58)	
75-84	1928 (28.25)	2458 (27.63)		510 (10.70)	269 (11.69)	
≥85	846 (12.40)	963 (10.82)		60 (1.26)	43 (1.87)	
Gender, No. (%)						
Male	4148 (60.78)	5439 (61.13)	.65	2664 (55.87)	1265 (54.95)	.47
Female	2677 (39.22)	3458 (38.87)		2104 (44.13)	1037 (45.05)	
Year						
2011	602 (8.82)	-		-	-	-
2012	725 (10.62)	-		-	-	-
2013	932 (13.66)	-	<.01	779 (16.34)	-	<.01
2014	917 (13.44)	200 (2.25)		853 (17.89)	3 (0.13)	
2015	675 (9.89)	824 (9.26)		949 (19.90)	21 (0.91)	
2016	607 (8.89)	1180 (13.26)		848 (17.79)	77 (3.34)	
2017	720 (10.55)	1892 (21.27)		443 (9.29)	585 (25.41)	
2018	887 (13.00)	2277 (25.59)		428 (8.98)	703 (30.54)	
2019	760 (11.14)	2524 (28.37)		468 (9.82)	913 (39.66)	
Laterality						
Right	3315 (48.57)	4412 (49.59)	<.01	1857 (38.95)	962 (41.79)	<.01
Left	3228 (47.30)	4199 (47.20)		1967 (41.25)	950 (41.27)	
Binocular	282 (4.13)	286 (3.21)		944 (19.80)	390 (16.94)	
Ophthalmology comorbidity, No. (%)						
Retinal neovascular	208 (3.05)	263 (2.96)	.74	14 (0.29)	8 (0.35)	.70
Central retinal vein occlusion	56 (0.82)	85 (0.96)	.37	64 (1.34)	48 (2.09)	.02
Branch retinal vein occlusion	97 (1.42)	142 (1.60)	.37	38 (0.80)	37 (1.61)	<.01
Charlson Comorbidity Index						
Mean	1.16 (1.66)	1.16 (1.68)	.96	3.25 (1.85)	3.61 (1.66)	<.01
0	3355 (49.16)	4436 (49.86)	.73	110 (2.31)	31 (1.35)	<.01
1	1558 (22.83)	1967 (22.11)		677 (14.20)	66 (2.87)	
2	714 (10.46)	928 (10.43)		749 (15.71)	302 (13.12)	
3+	1198 (17.55)	1566 (17.60)		3232 (67.79)	1903 (82.67)	
Co- ophthalmological medication, No. (%)						
Antibiotics						
Sulfonamides	827 (12.12)	767 (8.62)	<.01	485 (10.17)	206 (8.95)	.10
Fluoroquinolones	537 (7.87)	731 (8.22)	.43	413 (8.66)	167 (7.25)	.04
Others	1680 (24.62)	2323 (26.11)	.03	1444 (30.29)	611 (26.54)	<.01
Steroid/Antibiotic combination	1216 (17.82)	1608 (18.07)	.68	890 (18.67)	383 (16.64)	.04
Steroid	727 (10.65)	1112 (12.50)	<.01	348 (7.30)	124 (10.21)	<.01

Abbreviations: SD, standard deviation; wAMD, wet age-related macular degeneration; DME, diabetic macular edema.

 The distribution of laterality was comparable between the right and left eyes, with a lower proportion of binocular users. The rates of ophthalmic comorbidities, mainly related to other indications for reimbursed anti-VEGF treatments, were also analyzed, revealing a higher prevalence of ophthalmic diseases in aflibercept users than in ranibizumab users in general. DME patients, on average, showed a higher Charlson Comorbidity Index score (ranibizumab: 3.25 ± 1.85; aflibercept: 3.61 ± 1.66) than wAMD patients (ranibizumab: 1.16 ± 1.66; aflibercept: 1.16 ± 1.68). Moreover, approximately 60% of the study subjects received concomitant prescriptions of ophthalmological antibiotics.

 Student’s *t* tests were used for the comparisons of continuous variables, and χ^2^/Fisher’s tests were used for categorical variables.

###  Use of Anti-vascular Endothelial Growth Factor

**Figure 2:**
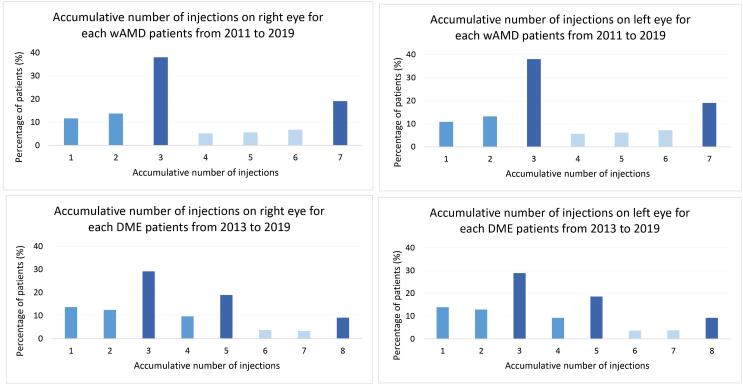
Accumulative Injection Numbers of Each Patient. Abbreviations: wAMD, wet age-related macular degeneration; DME, diabetic macular edema.

 Our study subjects received a total of 208 400 needles of anti-VEGF agents. After adopting the algorithm to label the administrative route of each injection, 105 244 needles were identified. After excluding obvious misclassification (patients had more needles than reimbursement limitations) and unknown sex, the study included 91 573 needles of anti-VEGF agents for further analyses (wAMD: 53.2%; DME: 32.1%) (Figure S2). In the analysis of the accumulative injection numbers of each patient, we found similar results in both wAMD and DME (Figure 2). First, most eyes received only three needles in total (wAMD: 38%; DME: 29%); Second, there was a higher proportion of needles with maximum limitations, seven for wAMD and eight for DME; Third, the proportions of needles within first application limitation were higher (wAMD: 1-3 needles; DME: 1-5 needles).

###  Interrupted Time Series Analysis 

**Figure 3:**
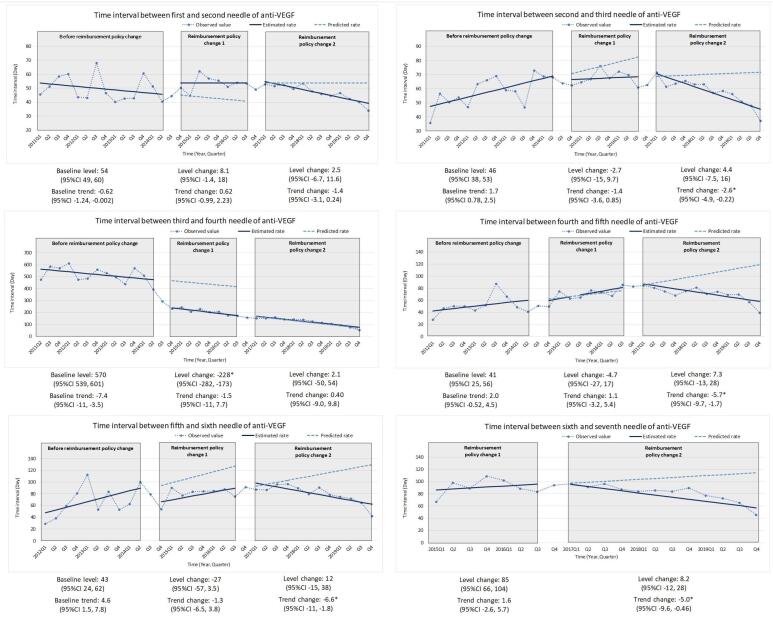
Quarterly Change of the Treatment Gap Between Each Anti-VEGF Injection Based on Reimbursement Criteria Among wAMD Patients (*P < .05). Abbreviations: CI, confidence interval; wAMD, wet age-related macular degeneration; anti-VEGF, anti-vascular endothelial growth factor.

**Figure 4:**
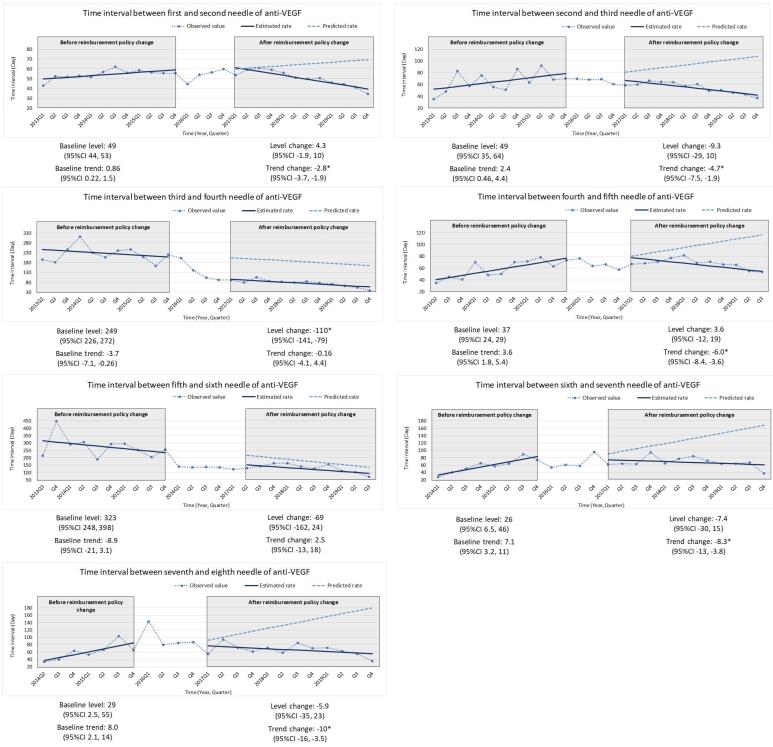
Quarterly Change of the Treatment Gap Between Each Anti-VEGF Injection Based on Reimbursement Criteria Among DME Patients (*P < .05). Abbreviations: CI, confidence interval; DME, diabetic macular edema; anti-VEGF, anti-vascular endothelial growth factor.

 Overall, regardless of indications, we found a decreasing trend in the treatment gap between each needle from 2011 to 2019. Among wAMD patients, our study found different effects of two types of reimbursement criteria change, cancellation of annual limitation (2014Q3) and prolongation of the usage period for each application (2016Q4) (Figure 3, Table S2, and Table S3). When the number of injections loosened from annual limitation to application limitation, there was a significant change in level in the treatment gap between the third and fourth needles (-228 days [95% CI -282 days, -173 days]). As for the second criteria revision, change of usage period, most treatment gaps showed a significant decreasing trend, except for the gap between the first and second and the gap between the third and fourth needles. A similar level change was found in DME patients (Figure 4, Table S2, and Table S3). There was a significant decrease in the treatment gap between the third and fourth needles (-110 days [95% CI -141 days, -79 days]). The treatment gap between the fifth and sixth needles expressed a similar pattern but without significant change. Other treatment gaps revealed significant change in slope after criteria changes.

## Discussion

 To the best of our knowledge, this is the first nationwide study using ITSA to demonstrate the impact of reimbursement policy on prescription patterns of anti-VEGF. The present study showed that changes in reimbursement criteria significantly impact the prescription patterns for both wAMD and DME patients. All treatment gaps exhibited a decreasing pattern throughout the observation period. The cancellation of the annual limitation had an immediate shortening effect on the treatment gap between the third and fourth injections. Additionally, the change in the usage period resulted in a long-term decrease in the treatment gap for subsequent injections. Furthermore, this study introduced a novel method for evaluating anti-VEGF injection sites. Specifically, the administration routes of co-ophthalmological medications were used to distinguish the injection sites of anti-VEGF treatments. This algorithm ensured a sufficient number of injections for analysis and revealed an expected distribution of the number of injections.

 The significantly dropped treatment gaps after removing the annual limitation might reflect the shortened treatment gap between three initial monthly injections and pro re nata injections (preferred for anti-VEGF treatment in routine practice over the fixed monthly injections used in clinical trials) in real-world practice. The change was also found in a retrospective study^13^ from the National Taiwan University Hospital database. The study included two groups of DME patients, from July 2013 to January 2015 (group A) and April 2016 to June 2017 (group B). The mean number of injections significantly increased from 4.6 ± 2.0 to 6.5 ± 2.3 in one year. It revealed that the 5-needle annual limitation on group A was highly related to utilization patterns. Under yearly restrictions, the treatment gap between initial monthly injections and pro re nata injections was prolonged which is aligned with our study results.

 Furthermore, a shortened treatment gap for injections has been proved to improve visual outcomes by numerous studies. Lai and colleagues showed a relationship between utilization changes and clinical outcomes.^13^ In correlation with the injection numbers, the mean improvement in best-corrected visual acuity at one year also revealed a more remarkable outcome (Group A: 5.8 letters; Group B: 14.8 letters), which implied that healthcare policy regulation change significantly influenced the visual outcomes of patients. Another Taiwanese retrospective cohort study^25^ also showed a comparable result. It included medical records from 5 hospital ophthalmology clinics and found that patients with DME who received more injections in 1 year could reach a better mean change in central macular thickness. Likewise, large phase III/IV clinical trials,^10,26^ in which patients received an average of seven to ten intravitreal injections per eye in the first year, have shown that frequent anti-VEGF injections maximize the improvement of visual and anatomic outcomes, including those of Asian population.

 As for the distribution pattern of the number of injections, we found a higher proportion of needles related to maximum limitations, seven for wAMD and eight for DME. Also, the proportions of needles within the first application limitation were higher (wAMD: 1-3 needles; DME: 1-5 needles). These results were consistent with several previous studies.^25,27,28^ Curtis et al found that Medicare beneficiaries who received any anti-VEGF injection for wAMD were treated with a mean number of 4.3 injections within a year. The distribution of the number of injections showed peaks at 1 and 3. Moreover, the data from Taiwan’s local study^25^ revealed a mean number of 3.72 injections administered in 1 year, with 69% of eyes receiving one to four anti-VEGF injections. Furthermore, the injection frequency reduced dramatically after the first quarter. Based on previous studies, we can further regard co-medications as a helpful method to interpret injection sites of anti-VEGF treatments.

 Another meaningful message from our study results is that annual limitation acts the most important role on impacting prescriptions. Over the last decade, the reimbursement criteria for anti-VEGF treatments have undergone several revisions, demanding considerable time and effort. The findings of this study could serve as a reference for shaping future policies, aiming to prevent unnecessary amendments to criteria. Apart from the changes on prescribing patterns, our study also found 60% patients received concomitant ophthalmological antibiotics after receiving intravitreal injection. However, numerous previous studies have suggested to avoid from this practice pattern, as it does not confer any benefits but rather contributes to the escalation of antibiotic resistance.^29,30^ Our study reveals this inappropriate prescription pattern and the potential of antibiotic resistance concerns on wAMD and DME patients receiving anti-VEGF treatments. Furthermore, the present study also observed a gradual decline in ranibizumab usage over the nine-year period. This trend can be attributed to the increased adoption of aflibercept since its introduction to the market in 2014. Initially, ranibizumab experienced a steady rise in use from 2011 to 2013. However, the introduction of aflibercept marked a turning point, leading to a noticeable decrease in ranibizumab utilization.

 Our study has several strengths. First, the use of ITSA, an uncontrolled, repeated cross-sectional design for collecting longitudinal data at an aggregate level, significantly reduces the risk of selection bias and minimizes confounding due to differences between groups (Table S4). Second, we ensured the inclusion of sufficient time points to accurately characterize trends and patterns, enhancing the reliability of our findings. Third, utilizing data from the NHI, a mandatory, government-operated, single-payer health insurance system that covers over 99% of Taiwan’s population, minimizes the potential for bias due to missing data. Additionally, the administration of anti-VEGF injections is confined to medical institutions, preventing self-administration by patients at home, which in turn reduces the likelihood of measurement misclassification in the treatment gaps between injections (the outcome of our study). Lastly, recognizing that the precise time point of “interruption” may not align with the immediate implementation of all intervention features, we have meticulously adjusted our analysis to account for the transition period, thereby addressing potential biases in the classification of the intervention.

 Despite all efforts spent on this study, there were still some limitations. First, we used the administration routes of co-ophthalmological medications to label the injection sites, which may cause misclassification of exposure. However, as part of the administration routes of anti-VEGF was reported as intravitreal injection, and could not ascertain the injection sites of treatment. We chose common co-medications to symbolize the injection eyes to avoid the small sample size of the study, and we found our results were comparable to numerous previous studies. Second, the study may include prior self-paid users. The first injection record of each patient during our observation period was regarded as their first needle, while they may have received previous injections out of pocket. In that case, the number of injections would be mislabeled. However, as ranibizumab and aflibercept were only available in 2011 in Taiwan, we considered this issue would not pose a great concern. Third, the results lack a direct relationship between clinical outcomes and utilization patterns. To gain enough study population, we use NHI claims data which clinical outcomes are unavailable. Future studies are warranted to discover the association between injection interval and visual outcomes.

## Conclusion

 This is the first nationwide study using ITSA to demonstrate the impact of reimbursement policy on treatment gaps between each anti-VEGF injection. We found that after canceling the annual three needles limitation, the treatment gaps significantly decreased among wAMD and DME patients. The shortened treatment gaps might further link to better visual outcomes according to previous studies. The different impacts from criteria changes can assist future policy shaping. Future studies were warranted to explore whether such changes are associated with the benefits of visual effects.

## Acknowledgments

 We thank the National Health Insurance Administration (NHIA) and HWDC for making the databases used in this study available; however, the content of this article does not represent any official position of the NHIA or HWDC. The authors had full access to all of the data in this study and take responsibility for the integrity of the data and accuracy of the data analysis.

## Ethical issues

 The Institutional Review Board of the National Taiwan University Hospital approved this study (202102015RIND), and informed consent was waived because of the de-identification nature of the study data.

## Competing interests

 Authors declare that they have no competing interests.

## Supplementary files

Supplementary file 1 contains Tables S1-S4 and Figures S1-S2.
