# Supplementary material for: Changing Reimbursement Criteria on Anti-VEGF Treatment Patterns Among Wet Age-Related Macular Degeneration and Diabetic Macular Edema Patients: An Interrupted Time Series Analysis
Source: Int J Health Policy Manag. 2024 Jul 7;13:8210. doi: 10.34172/ijhpm.8210 (PMC11365169; doi:10.34172/ijhpm.8210)

Article title: Changing Reimbursement Criteria on Anti-VEGF Treatment Patterns Among Wet Age-Related Macular Degeneration and Diabetic Macular Edema Patients: An Interrupted Time Series Analysis

Journal name: International Journal of Health Policy and Management (IJHPM)

Authors' information: Yu-Wen Huang[1]¶, Lin-Chieh Meng[2]¶, Li-Jiuan Shen[1,2,3], Chih-Fen Huang[1,3], Liang-Kung Chen[4,5,6], Fei-Yuan Hsiao[1,2,3]*

[1]School of Pharmacy, National Taiwan University, Taipei, Taiwan.

[2]Graduate Institute of Clinical Pharmacy, College of Medicine, National Taiwan University, Taipei, Taiwan.

[3]Department of Pharmacy, National Taiwan University Hospital, Taipei, Taiwan.

[4]Center for Geriatrics and Gerontology, Taipei Veterans General Hospital, Taipei, Taiwan.

[5]Center for Healthy Longevity and Aging Sciences, National Yang Ming Chiao Tung University, Hsinchu, Taiwan.

[6]Taipei Municipal Gan-Dau Hospital (Managed by Taipei Veterans General Hospital), Taipei, Taiwan.

***Correspondence to:** Fei-Yuan Hsiao; Email: fyshsiao@ntu.edu.tw

¶Both authors contributed equally to this paper

Citation: Huang YW, Meng LC, Shen LJ, Huang CF, Chen LK, Hsiao FY. Changing reimbursement criteria on antiVEGF treatment patterns among wet age-related macular degeneration and diabetic macular edema patients: an interrupted time series analysis. Int J Health Policy Manag. 2024;13:8210. doi:10.34172/ijhpm.8210

Supplementary file 1

Table S1. Reimbursement criteria changes of anti-VEGF in Taiwan

wAMD	
January 2011 - July 2014	• Patients ≧50 y/o.<br>• A maximum of 3 intravitreal injections are reimbursed for each year<br>• The usage period is limited to 2 years.
August 2014 - November 2016	• Application limited to one Anti-VEGF treatment (Ranibizumab or Aflibercept)<br>• Patients ≧50 y/o.<br>• A maximum of 3 intravitreal injections are reimbursed for the first application. Maximum 7 needles.<br>• The usage period is limited to 2 years for each application.
December 2016 - May 2020	• Application limited to one Anti-VEGF treatments (Ranibizumab or Aflibercept)<br>• Patients ≧50 y/o.<br>• A maximum of 3 intravitreal injections are reimbursed for the first application. Maximum 7 needles.<br>• The usage period is limited to 5 years for each application.
DME	
Feburary 2013 - January 2016	• A maximum of 5 intravitreal injections are reimbursed for the first year.<br>• A maximum of 3 intravitreal injections are reimbursed for the second year.

Feburary 2016<br>- October 2016	• A maximum of 5 intravitreal injections are reimbursed for the first application. Maximum 8 needles.<br>• The usage period is limited to 2 years for each application.
November 2016<br>- November 2016	• Application limited to one Anti-VEGF treatment (Ranibizumab or Aflibercept)<br>• A maximum of 5 intravitreal injections are reimbursed for the first application. Maximum 8 needles.<br>• The usage period is limited to 2 years for each application.
December 2016	• Application limited to one Anti-VEGF treatment (Ranibizumab or Aflibercept)<br>• A maximum of 5 intravitreal injections are reimbursed for the first application. Maximum 8 needles.<br>• The usage period is limited to 5 years for each application.

Table S2. Results in change of the treatment gap between each anti-VEGF injection based on reimbursement criteria: interrupted time series analyses.

	wAMD		DME
	**Policy change 1**	**Policy change 2**	**Policy change**
1st- 2nd	x	x	Significant trend change
2nd- 3rd	x	Significant trend change	Significant trend change
3rd- 4th	Significant level change	x	Significant level change
4th- 5th	x	Significant trend change	Significant trend change
5th- 6th	x	Significant trend change	x
6th- 7th	x	Significant trend change	Significant trend change
7th- 8th	-	-	Significant trend change

x: No significant change; -: No results

Table S3. Results in change of the treatment gap between each anti-VEGF injection based on reimbursement criteria: interrupted time series analyses.

(A) Prescription gap of anti-VEGF before and after reimbursement policy change for wAMD patients

Statistical methods:

$Y = \beta_0 + \beta_1$ (time)+ β_2 (Reimbursement policy change 1)+ β_3 (Time after change 1)+ β_4 (Reimbursement policy change 2)+ β_5 (Time after change 2)+ e_i

Y	Study outcome: the treatment gap between each injection by days.
Time	A continuous variable indicating time in quarters from the start of reimbursement date(Time= 2011Q1) to the end of observation period (Time= 2019Q4)
Reimbursement policy change 1	A variable indicating the reimbursement policy change 1 (coded 0 for period before Reimbursement policy change 1; coded 1 for period after reimbursement policy change 1)
Time after change 1	A continuous variable indicating time in quarters after the reimbursement policy change 1 (coded 0 before the Reimbursement policy change 1; coded 1 to 8 after the reimbursement policy change 1)
Reimbursement policy change 2	A variable indicating the reimbursement policy change 2 (coded 0 for period before the Reimbursement policy change 2; coded 1 for period after the reimbursement policy change 2)
Time after change 2	A continuous variable indicating time in quarters after the reimbursement policy change 2 (coded 0 before the reimbursement policy change 2; coded 1 to 12 after the reimbursement policy change 2)
$\beta 0$	Estimates the "baseline level" of the study outcome before reimbursement policy changes (the intercept)
$\beta 1$	Estimates the "baseline trend" of the study outcome before reimbursement policy changes (the slope)
$\beta 2$ and $\beta 4$	Estimates the "level change" of the study outcome at each reimbursement policy change (an immediate change)
$\beta 3$ and $\beta 5$	Estimates the "trend change" of the study outcome between each reimbursement policy change (difference between the slopes)

Results:

Prescription gap	Intercept (95% CI)	Slope (95% CI)	Reimbursement policy change 1 Absolute effects		Reimbursement policy change 2 Absolute effects	
			Change in level (95% CI)	Change in slope (95% CI)	Change in level (95% CI)	Change in slope (95% CI)
between first and second	54 (49 to 60)	-0.62 (-1.24 to -0.002)	8.1 (-1.4 to 18)	0.62 (-0.99 to 2.23)	2.5 (-6.7 to 11.6)	-1.4 (-3.1 to 0.24)
between second and third	46 (38 to 53)	1.7 (0.78 to 2.5)	-2.7 (-15 to 9.7)	-1.4 (-3.6 to 0.85)	4.4 (-7.5 to 16)	-2.6 (-4.6 to -0.22)[b]
between third and fourth	570 (539 to 601)	-7.4 (-11 to -3.5)	-228 (-282 to -173)[b]	-1.5 (-11 to 7.7)	2.1 (-50 to 54)	0.40 (-9.0 to 9.8)
between fourth and fifth	41 (25 to 56)	2.0 (-0.52 to 4.5)	-4.7 (-27 to 17)	1.1 (-3.2 to 5.4)	7.3 (-13 to 28)	-5.7 (-9.7 to -1.7)[b]
between fifth and sixth	43 (24 to 62)	4.6 (1.5 to 7.8)	-27 (-57 to 3.5)	-1.3 (-6.5 to 3.8)	12 (-15 to 38)	-6.6 (-11 to -1.8)[b]
between sixth and seventh	N/A[a]	N/A[a]	85 (66 to 104)	1.6 (-2.6 to -5.7)	8.2 (-12 to 28)	-5.0 (-9.6 to -0.46)[b]

a. The seventh needle was reimbursed after policy change 1.

b. $p < 0.05$

(B) Prescription gap of anti-VEGF before and after reimbursement policy change for DME patients

Statistical methods:

$$\mathbf{Y = \beta_0 + \beta_1\ (time) + \beta_2\ (Reimbursement\ policy\ change) + \beta_3\ (Time\ after\ change\ ) + e_i}$$

Y	Study outcome: the treatment gap between each injection by days.
Time	A continuous variable indicating time in quarters from the start of reimbursement date(Time= 2013Q1) to the end of observation period (Time= 2019Q4)
Reimbursement policy change	A variable indicating the reimbursement policy change 1 (coded 0 for period before reimbursement policy change 1; coded 1 for period after reimbursement policy change 1)
Time after change	A continuous variable indicating time in quarters after the reimbursement policy change 1 (coded 0 before the reimbursement policy change 1; coded 1 to 12 after the reimbursement policy change 1)
β0	Estimates the "baseline level" of the study outcome before reimbursement policy changes (the intercept)
β1	Estimates the "baseline trend" of the study outcome before reimbursement policy changes (the slope)
β2	Estimates the "level change" of the study outcome at the reimbursement policy change (an immediate change)
β3	Estimates the "trend change" of the study outcome after the reimbursement policy change (difference between the slopes)

Results:

Prescription gap	Intercept (95% CI)	Slope (95% CI)	Reimbursement policy change Absolute effects	
			Change in level (95% CI)	Change in slope (95% CI)
between first and second	49 (44 to 53)	0.86 (0.22 to 1.5)	4.3 (-1.9 to 10)	-2.8 (-3.7 to -1.9)[a]
between second and third	49 (35 to 64)	2.4 (0.46 to 4.4)	-9.3 (-29 to 10)	-4.7 (-7.5 to -1.9)[a]
between third and fourth	249 (226 to 272)	-3.7 (-7.1 to -0.26)	-110 (-141 to -79)[a]	-0.16 (-4.1 to 4.4)
between fourth and fifth	37 (24 to 29)	3.6 (1.8 to 5.4)	3.6 (-12 to 19)	-6.0 (-8.4 to -3.6)[a]
between fifth and sixth	323 (248 to 398)	-8.9 (-21 to 3.1)	-69 (-162 to 24)	2.5 (-13 to 18)
between sixth and seventh	26 (6.5 to 46)	7.1 (3.2 to 11)	-7.4 (-30 to 15)	-8.3 (-13 to -3.8)[a]
between seventh and eighth	29 (2.5 to 55)	8.0 (2.1 to 14)	-5.9 (-35 to 23)	-10 (-16 to -3.5)[a]
a. $p < 0.05$				

Table S4. Evaluation of the risk of bias in interrupted time series analysis (ITSA)

ITSA is a quasi-experimental method that can identify pre-existing trends in treatment gaps and estimate changes resulting from revisions in reimbursement criteria. However, several domains of bias, as outlined in the ROBINS-I tool (https://training.cochrane.org/handbook/current/chapter-25), may exist for ITSA as follows:

**Bias domain**	**Issues addressed in our study**
• Bias due to confounding	The ITSA utilized an uncontrolled, repeated cross-sectional design to collect longitudinal data measured at an aggregate level. This approach minimizes confounding due to between-group differences, offering a significant advantage. In our study, beyond the changes in reimbursement criteria for anti-VEGF treatments, there is a minimal possibility that extraneous events or context changes occurred around the time the intervention was introduced. We also ensured the inclusion of sufficient time points to accurately characterize trends and patterns (for example, the segmented regression models for wAMD comprised a total of 14 data points before the criteria change (2011 Q1–2014 Q2), 7 data points after the first criteria change (2014 Q4–2016 Q3), and 12 data points subsequent to the second criteria change (2017 Q1–2019 Q4)). Furthermore, to address autocorrelation within individual data points, we applied the Durbin-Watson test, enhancing the robustness of our findings. This methodology aligns with the ROBINS-I evaluation criteria for an uncontrolled before-after study, providing a sound basis for our analysis.
• Bias in selection of participants into the study<br>• Bias due to missing data<br>• Bias in measurement of the outcome	Our study conducts a retrospective, nationwide analysis using data from the NHI program. The NHI is a mandatory, government-operated, single-payer health insurance system that covers over 99% of Taiwan's population, approximately 24 million people. It offers comprehensive coverage, including outpatient and inpatient services, medications, diagnostic tests, procedures, and surgeries for its beneficiaries. The system meticulously records detailed healthcare information for each beneficiary, covering demographics, healthcare utilization, diagnoses, procedures, and drug prescriptions. Therefore, the potential for bias due to missing data is minimal. Moreover, ITSA sets itself apart from most other intervention study designs by utilizing a before-after comparison within a single population, rather than comparing to a control group. This method significantly reduces the risk of selection bias, thereby ensuring a more robust analysis of the intervention's impact within the NHI framework. Additionally, the administration of anti-VEGF injections is confined to medical institutions and cannot be self-administered by patients at home. Thus, the treatment gaps between injections (the outcome in our study) are less susceptible to measurement misclassification.
• Bias in classification of interventions<br>• Bias due to deviations from intended interventions<br>• Bias in selection of the reported result	Recognizing that the exact time point of 'interruption' may not coincide with the immediate implementation of all intervention features, we have carefully adjusted our analysis to consider these nuances. For the wAMD segmented regression model, we excluded two data points within the transition period (Q3 2014 and Q4 2016) to mitigate potential biases arising from the timing of intervention classification. Similarly, for DME, we excluded data from four points during the transition period (Q1–Q4 2016). This decision aims to prevent erroneously attributing observed effects solely to the intervention, without accounting for the gradual implementation or the presence of intervention effects before or after its intended full-scale implementation. By doing so, we can address potential biases in the classification of the intervention and in the selection of reported results.

Figure S1. Illustration of study hypothesis and outcome of interest (treatment gap between injections)

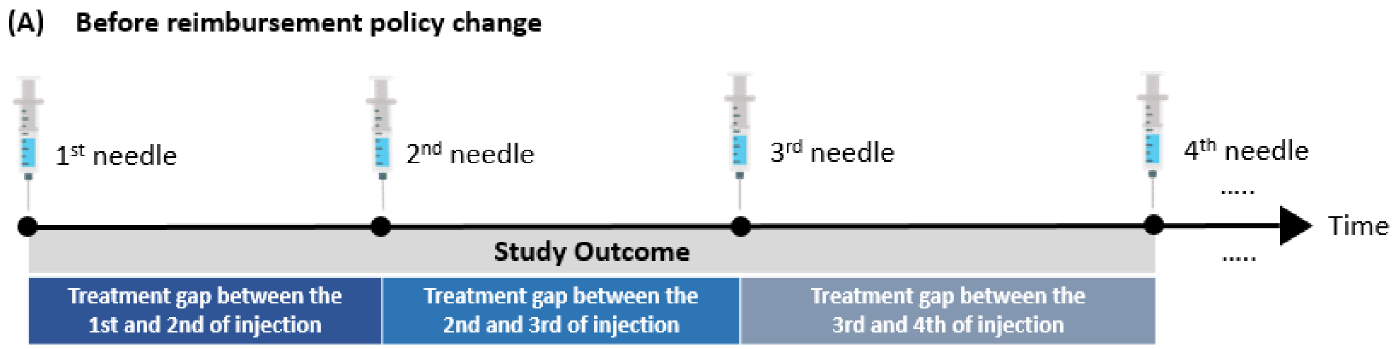

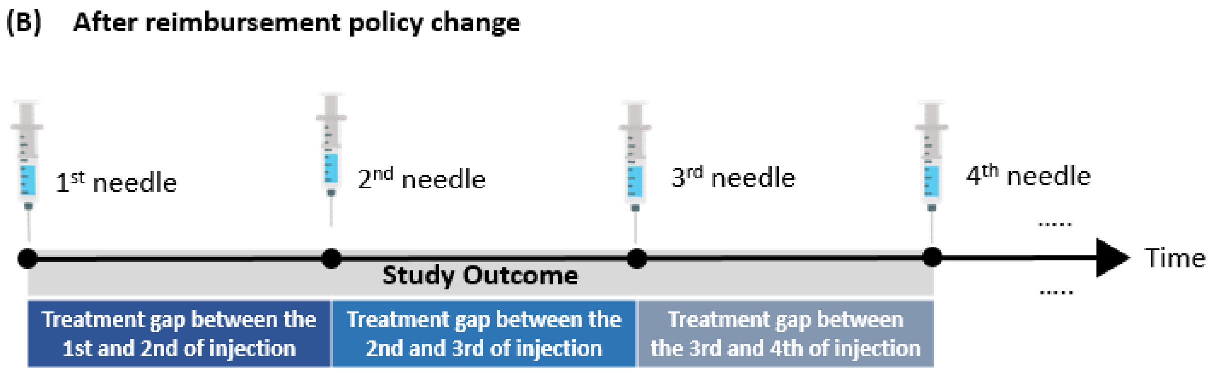

Figure S2. Flowchart of study cohort

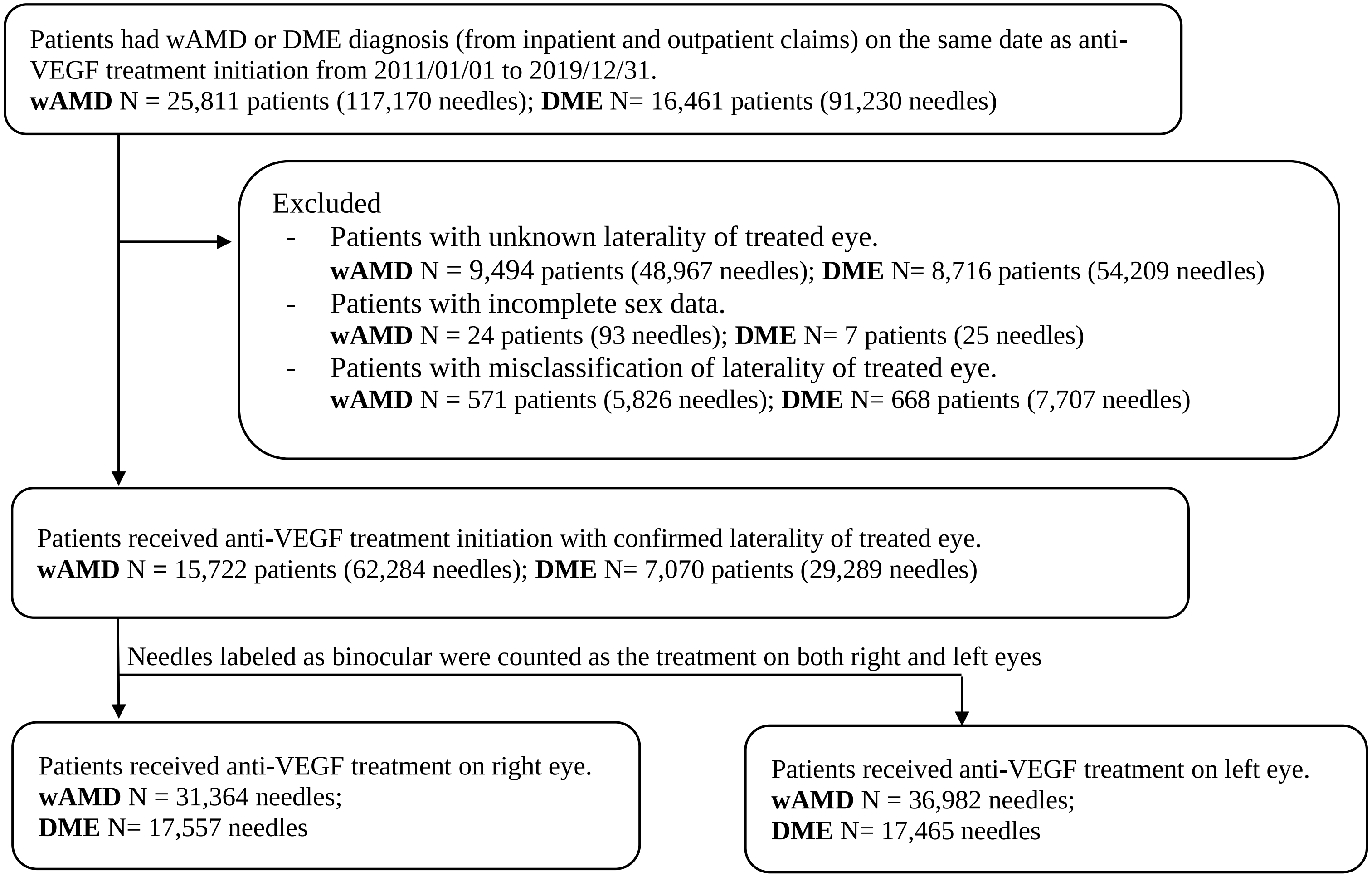

Supplement: Supplementary file 1 — contains Tables S1-S4 and Figures S1-S2. [file ijhpm-13-8210-s001.pdf]
